# Complete Genome Sequences of Diverse Uropathogenic Staphylococcus saprophyticus Isolates from a College Health Center

**DOI:** 10.1128/MRA.00722-20

**Published:** 2020-08-27

**Authors:** Craig Stephens, Megan Wright, Austin Hartman, Andres Gonzalez, George Sensabaugh, Peggie Robinson, David Hess

**Affiliations:** a Biology Department, Santa Clara University, Santa Clara, California, USA; b Public Health Program, Santa Clara University, Santa Clara, California, USA; c Department of Computer Science, Santa Clara University, Santa Clara, California, USA; d School of Public Health, University of California, Berkeley, Berkeley, California, USA; e Cowell Student Health Services, Santa Clara University, Santa Clara, California, USA; Loyola University Chicago

## Abstract

Staphylococcus saprophyticus is a significant cause of urinary tract infections in younger women, but it has been understudied at the genomic level. We report genome sequences of six S. saprophyticus isolates obtained from female patients who presented with urinary tract infection symptoms at a college health center in 2019.

## ANNOUNCEMENT

Staphylococcus saprophyticus is a common coagulase-negative *Staphylococcus* species found on the skin of humans (1), and it is the second most frequent bacterial cause of acute uncomplicated urinary tract infections in younger women (2–4). This species has been poorly studied at the genomic level, relative to other staphylococci, with only one fully assembled genome sequence described in print (strain ATCC 15305) (5). Further analyses enabled by the genomes presented here will facilitate understanding the genetic diversity of this species and determinants of S. saprophyticus pathogenesis.

The six isolates presented here were obtained in 2019 from female patients at the Santa Clara University Cowell Student Health Center who presented with symptoms of urinary tract infection. The study protocol and informed consent documents were approved by the Human Subjects Research Committee at Santa Clara University. Urine was streaked onto CHROMagar Orientation agar (6). These isolates were subsequently identified as S. saprophyticus by biochemical testing and 16S rRNA sequencing. Genomic DNA was prepared from Luria-Bertani broth cultures grown overnight at 37°C, pelleted, and quick frozen. For short-read sequencing, DNA was prepared using the Quick-DNA fungal/bacterial micropreparation kit (Zymo Research). Libraries were prepared with the Nextera DNA Flex library preparation kit (Illumina) and analyzed on an Illumina MiniSeq instrument using two-channel sequencing-by-synthesis chemistry and 150-base paired-end reads. Reads were trimmed for quality and adapters using the BBDuk plugin for Geneious v11.1.5 (Biomatters Ltd.). For long-read sequencing, genomic DNA was prepared with the NucleoSpin microbial DNA kit (Macherey-Nagel). Libraries were prepared by barcoding native genomic DNA with the ligation sequencing kit (SQK-LSK109; Oxford Nanopore Technologies) and were analyzed with a MinION SpotON flow cell (FLO-MIN106D, R9 version). Raw data were processed by the Guppy basecaller v3.2.10 in MinKNOW v3.6.5. Demultiplexing and trimming of barcodes were performed through the Epi2Me portal. Assembly was performed with Unicycler v0.4.8 (7) using default hybrid assembly settings, which confirmed the circularity of the contigs. Annotation was performed with the NCBI Prokaryotic Genome Annotation Pipeline v4.11 (8).

Chromosomes of all isolates were aligned with S. saprophyticus ATCC 15305 (GenBank accession number AP008934) using Mauve (9). No major rearrangements were detected; small alignment gaps were associated with distinct staphylococcal chromosome cassettes (SCCs) at the *iss* site (10) and a small number of prophages. The isolates contained one to four plasmids, ranging in size from 2.2 to 74 kb. Using BLAST (11) and Mauve (9), only pUTI-050-2 aligned with a plasmid from ATCC 15305, pSSP1.

### Data availability.

All sequences and read data have been deposited in GenBank and the Sequence Read Archive (SRA) under the accession numbers shown in Table 1. These files are part of BioProject PRJNA636387.

**TABLE 1:** *S. saprophyticus* isolate genomes and GenBank accession numbers

Parameter	Data for isolate:					
	UTI-035	UTI-042y	UTI-045	UTI-050	UTI-056	UTI-058y
No. of reads used in final assembly^*a*^	I: 1,594,360; N: 63,161	I: 1,180,522; N: 49,369	I: 1,549,872; N: 20,661	I: 1,211,292; N: 25,302	I: 1,477,810; N: 36,579	I: 1,656,358; N: 26,363
Nanopore read *N*_50_ (bp)	8,375	8,231	19,958	17,136	15,963	17,573
Read coverage (×)	I: 84; N: 105	I: 60; N: 78	I: 77; N: 96	I: 62; N: 106	I: 78; N: 145	I: 87; N: 113×
SRA accession no.	I: SRR12056308; N: SRR12057713	I: SRR12056053; N: SRR12057712	I: SRR12056174; N: SRR12057711	I: SRR12056166; N: SRR12057710	I: SRR12056163; N: SRR12057709	I: SRR12056311; N: SRR12057708
Chromosome						
Size (bp)	2,569,353	2,645,137	2,639,927	2,601,138	2,553,871	2,550,339
Accession no.	CP054434	CP054438	CP054831	CP054575	CP054444	CP054440
Plasmid(s) (size [bp], accession no.)	pUTI-035-1 (36,907, CP054435);	pUTI-042y-1 (2,305, CP054439)	pUTI-045-1 (74,014, CP054832);	pUTI-050-1 (41,251, CP054576);	pUTI-056-1 (39,196, CP054445);	pUTI-058y-1 (42,714, CP054441);
	pUTI-035-2 (35,615, CP054436);		pUTI-045-2 (19,933, CP054833);	(pUTI-050-2 (29,715, CP054577);	pUTI-056-2 (36,906, CP054446);	pUTI-058y-2 (36,907, CP054442);
	pUTI-035-3 (2,458, CP054437)		pUTI-045-3 (8,128, CP054834);	pUTI-050-3 (28,716, CP054578)	pUTI-056-3 (4,439, CP054447);	pUTI-058y-3 (2,276, CP054443)
			pUTI-045-4 (2,276, CP054835)	pUTI-050-4 (2,276, CP054579)	pUTI-056-4 (2,276, CP054448)	
Genome GC content (%)	33.1	33.2	33.1	33.2	33.1	33.1

a I, Illumina; N, Nanopore.

## References

[1] Becker K, Heilmann C, Peters G 2014 Coagulase-negative staphylococci. Clin Microbiol Rev 27:870–926. doi:10.1128/CMR.00109-13. 25278577 PMC4187637

[2] Jordan PA, Iravani A, Richard GA, Baer H 1980 Urinary tract infection caused by *Staphylococcus saprophyticus*. J Infect Dis 142:510–515. doi:10.1093/infdis/142.4.510. 7192302

[3] Marrie TJ, Kwan C, Noble MA, West A, Duffield L 1982 *Staphylococcus saprophyticus* as a cause of urinary tract infections. J Clin Microbiol 16:427–431. doi:10.1128/JCM.16.3.427-431.1982. 6890071 PMC272384

[4] Eriksson A, Giske CG, Ternhag A 2013 The relative importance of *Staphylococcus saprophyticus* as a urinary tract pathogen: distribution of bacteria among urinary samples analysed during 1 year at a major Swedish laboratory. APMIS 121:72–78. doi:10.1111/j.1600-0463.2012.02937.x. 23030816

[5] Kuroda M, Yamashita A, Hirakawa H, Kumano M, Morikawa K, Higashide M, Maruyama A, Inose Y, Matoba K, Toh H, Kuhara S, Hattori M, Ohta T 2005 Whole genome sequence of *Staphylococcus saprophyticus* reveals the pathogenesis of uncomplicated urinary tract infection. Proc Natl Acad Sci U S A 102:13272–13277. doi:10.1073/pnas.0502950102. 16135568 PMC1201578

[6] Samra Z, Heifetz M, Talmor J, Bain E, Bahar J 1998 Evaluation of use of a new chromogenic agar in detection of urinary tract pathogens. J Clin Microbiol 36:990–994. doi:10.1128/JCM.36.4.990-994.1998. 9542923 PMC104675

[7] Wick RR, Judd LM, Gorrie CL, Holt KE 2017 Unicycler: resolving bacterial genome assemblies from short and long sequencing reads. PLoS Comput Biol 13:e1005595. doi:10.1371/journal.pcbi.1005595. 28594827 PMC5481147

[8] Tatusova T, DiCuccio M, Badretdin A, Chetvernin V, Nawrocki EP, Zaslavsky L, Lomsadze A, Pruitt KD, Borodovsky M, Ostell J 2016 NCBI Prokaryotic Genome Annotation Pipeline. Nucleic Acids Res 44:6614–6624. doi:10.1093/nar/gkw569. 27342282 PMC5001611

[9] Darling AC, Mau B, Blattner FR, Perna NT 2004 Mauve: multiple alignment of conserved genomic sequence with rearrangements. Genome Res 14:1394–1403. doi:10.1101/gr.2289704. 15231754 PMC442156

[10] Wang L, Safo M, Archer GL 2012 Characterization of DNA sequences required for the CcrAB-mediated integration of staphylococcal cassette chromosome *mec*, a *Staphylococcus aureus* genomic island. J Bacteriol 194:486–498. doi:10.1128/JB.05047-11. 22056931 PMC3256654

[11] Altschul SF, Gish W, Miller W, Myers EW, Lipman DJ 1990 Basic local alignment search tool. J Mol Biol 215:403–410. doi:10.1016/S0022-2836(05)80360-2. 2231712

